# Urinary glycoproteins associated with aggressive prostate cancer

**DOI:** 10.7150/thno.47066

**Published:** 2020-10-25

**Authors:** Mingming Dong, T. Mamie Lih, Shao-Yung Chen, Kyung-Cho Cho, Rodrigo Vargas Eguez, Naseruddin Höti, Yangying Zhou, Weiming Yang, Leslie Mangold, Daniel W. Chan, Zhen Zhang, Lori J. Sokoll, Alan Partin, Hui Zhang

**Affiliations:** 1 Department of Pathology, School of Medicine, Johns Hopkins University, Baltimore, Maryland 21231, USA.; 2 Department of Chemical and Biomolecular Engineering, Johns Hopkins University, Baltimore 21218, Maryland, USA.; 3 The Brady Urological Institute, The Johns Hopkins School of Medicine, Baltimore, Maryland 21287, USA.

**Keywords:** aggressive prostate cancer, urinary biomarkers, noninvasive prostate cancer, glycoproteomics, mass spectrometry

## Abstract

**Background:** There is an urgent need for the detection of aggressive prostate cancer. Glycoproteins play essential roles in cancer development, while urine is a noninvasive and easily obtainable biological fluid that contains secretory glycoproteins from the urogenital system. Therefore, here we aimed to identify urinary glycoproteins that are capable of differentiating aggressive from non-aggressive prostate cancer.

**Methods:** Quantitative mass spectrometry data of glycopeptides from a discovery cohort comprised of 74 aggressive (Gleason score ≥8) and 68 non-aggressive (Gleason score = 6) prostate cancer urine specimens were acquired via a data independent acquisition approach. The glycopeptides showing distinct expression profiles in aggressive relative to non-aggressive prostate cancer were further evaluated for their performance in distinguishing the two groups either individually or in combination with others using repeated 5-fold cross validation with logistic regression to build predictive models. Predictive models showing good performance from the discovery cohort were further evaluated using a validation cohort.

**Results:** Among the 20 candidate glycoproteins, urinary ACPP outperformed the other candidates. Urinary ACPP can also serve as an adjunct to serum PSA to further improve the discrimination power for aggressive prostate cancer (AUC= 0.82, 95% confidence interval 0.75 to 0.89). A three-signature panel including urinary ACPP, urinary CLU, and serum PSA displayed the ability to distinguish aggressive prostate cancer from non-aggressive prostate cancer with an AUC of 0.86 (95% confidence interval 0.8 to 0.92). Another three-signature panel containing urinary ACPP, urinary LOX, and serum PSA also demonstrated its ability in recognizing aggressive prostate cancer (AUC=0.82, 95% confidence interval 0.75 to 0.9). Moreover, consistent performance was observed from each panel when evaluated using a validation cohort.

**Conclusion:** We have identified glycopeptides of urinary glycoproteins associated with aggressive prostate cancer using a quantitative mass spectrometry-based glycoproteomic approach and demonstrated their potential to serve as noninvasive urinary glycoprotein biomarkers worthy of further validation by a multi-center study.

## Introduction

Prostate cancer (PCa) is the most frequently diagnosed cancer and the second leading cause of cancer-related death for men 1. However, most patients presenting with PCa actually have a low-risk form (Gleason score = 6) that does not require interventions including unnecessary biopsies and treatments 2. Currently, there are no Food and Drug Administration (FDA)-approved noninvasive biomarkers that can be used to differentiate aggressive (AG) from non-aggressive (NAG) PCa. Therefore, discovering noninvasive biomarkers for AG PCa is crucial.

Urine is a promising specimen for the discovery of noninvasive biomarkers associated with PCa. Urine-derived genetic biomarkers from RNA and DNA or metabolites have been investigated for their diagnostic and prognostic value for PCa 3-13. Urine-derived long non-coding RNA prostate cancer antigen-3 (PCA3) is the first US FDA approved urinary biomarker for PCa to aid decision making for repeated biopsies 14, while its ability in detecting the aggressiveness of PCa is limited 15. A recent multi-center study assessed the diagnostic and prognostic values of PCA3 and TMPRSS2:ERG gene fusions for PCa., in which TMPRSS2:ERG gene fusions, other than PCA3, has demonstrated prognostic value for PCa 7. Urinary miRNAs have been proposed as potential prognostic biomarkers for PCa and different panels of urinary miRNAs have been used to predict the aggressiveness of the cancer with comparable performance to most tissue-based prognostic assays (AUC around 0.7) 11. Besides miRNAs, urine proteins have also been investigated, for which moderate performance is observed in distinguishing the extracapsular stage pT3 PCa from the organ-confined stage pT2 PCa (AUC=0.74) 9. Nonetheless, due to the heterogeneous nature of PCa 6, there is still no ideal marker for the detection of aggressive PCa. Thus, it is essential to discover novel biomarkers that can work independently or in combination with currently available biomarkers to improve the discrimination power towards aggressive PCa.

Glycoproteins play essential roles in cancer development or progression 16-22. Most of the FDA-approved biomarkers for cancer diagnosis and monitoring are glycoproteins 16. They are often present on the cell surface or secreted from cells; therefore, they can be found in body fluids (e.g., serum or urine) and serve as noninvasive biomarkers. For the study of PCa, urinary glycoproteins are appealing targets for several reasons. First, the urine collected immediately after the digital rectal examination (DRE) of PCa patients may contain glycoproteins secreted or shed from tumor tissues that may be associated with the aggressiveness of the cancer. Second, our previous research has demonstrated that the majority of cancer-associated glycoproteins identified from prostate tissue samples are more readily detected in patients' urine in higher abundances than in their serum 23, 24. These studies have laid the foundation for the increased use of urine specimens to identify glycoprotein biomarkers for PCa. While high-throughput analysis of clinical specimens is essential for biomarker discovery, the low protein concentration and interfering compounds in urine make it quite challenging for high-throughput proteomic analysis. Recently, our lab has reported an automated sample preparation procedure to process urine samples for proteomics and glycoproteomics analysis with high reproducibility and high throughput 25, 26, which paves the way for analyzing large cohorts of clinical urine samples.

Furthermore, the rapid development of mass spectrometry (MS) technology has also advanced biomarker discovery. Data independent acquisition (DIA) MS works as a powerful tool offering high throughput and reproducibility for quantitative proteomics 27, which is suitable for analyzing large-scale clinical cohorts. Another advantage of DIA MS is that the acquired data set can be reprocessed to obtain previously unidentified features and make parallel comparisons of data acquired at different times. The acquired DIA data can serve as a digital bank of clinical samples, which would benefit long term biomarker screening and aid in the search for novel biomarkers based on the same samples without having to recollect the data. Therefore, DIA MS was used for quantitative analysis in this study because of its high-throughput, cost-effective and flexible nature.

In this study, a high-throughput and integrated workflow for urinary glycoproteomics analysis was employed. We used an automated approach for urine sample preparation and glycopeptide isolation 25, 26, coupled with DIA MS, to systematically and effectively conduct the quantitative analysis of glycopeptides derived from urine in order to discover unique urinary glycoproteins distinguishing aggressive PCa from non-aggressive PCa. We also compared and evaluated a combinatorial approach with candidate urinary glycopeptides and serum PSA, which may improve performance for aggressive PCa diagnosis.

## Methods

### Chemicals and reagents

C4 resin beads (35 μm, 300 Å) were purchased from Separation Methods Technologies (Newark, DE). Oasis MAX resins and Sep-Pak C18 resins were obtained from Waters (Milford, MA). Sequencing-grade trypsin and Lys-C were acquired from Promega (Madison, WI). Other chemicals including urea, ammonia bicarbonate (AB), acetonitrile (ACN), trifluoroacetic acid (TFA), triethyl ammonium bicarbonate (TEAB), tris (2-carboxyethyl) phosphine (TCEP), iodoacetamide, and triethylammonium acetate were purchased from Sigma Aldrich (St. Louis, MO). Indexed retention time (iRT) standards (a mixture of eleven peptides) were purchased from Biognosys Inc (Zurich, Switzerland).

### Automated tryptic digestion of human urine samples

A discovery cohort containing post-digital rectal examination (DRE) urine samples from 74 AG PCa patients (Gleason score ≥8) and 68 NAG PCa patients (Gleason score = 6) as well as a validation cohort consisting of 77 post-DRE urine samples (40 AG PCa and 37 NAG PCa) were collected by the Department of Urology at Johns Hopkins University School of Medicine with approval from the Institutional Review Board of Johns Hopkins University under informed consent. Detailed information on the clinical urine specimens is listed in Table S1.

The urine samples (500 µL) were desalted and protease digested on Versette (Thermo Scientific, Waltham, MA) according to the automated procedures that we published previously 25. In brief, each aspiration/dispense cycle was performed in approximately two minutes at room temperature. C4-tips were fabricated with 30 mg of C4 resin beads packed into each tip and conditioned with 50% ACN containing 0.1% TFA followed by 0.1% TFA (10 cycles each). Next, urine samples (500 μL) were acidified (pH < 3) and then loaded onto the C4-tips (90 aspiration/dispense cycles). The tips were rinsed with 0.1% TFA followed by 100 mM triethyl ammonium bicarbonate (TEAB) to remove unbound and contaminant material (10 cycles each). Proteins binding onto the C4-tips were reduced with 10 mM Tris 2-carboxyethyl phosphine (TCEP) in 50mM TEAB buffer (pH 8.2) at room temperature and alkylated with 15 mM iodoacetamide in the dark (20 cycles each). Proteins were digested (1:40 enzyme/protein) by Lys-C for one hour (30 cycles) followed by trypsin digestion for another six hours (120 cycles) in 50 mM TEAB buffer containing 30% ACN to directly recover digested peptides from C4-tips to solution. The C4-tips were subsequently rinsed twice with 50% ACN containing 0.1% TFA to elute the remaining digested peptides into the solution. Peptide mixtures were dried down and stored at -20 °C until analyzed.

### Isolation of N-linked glycosite-containing peptide from human urine samples

Intact glycopeptides were isolated from the peptide mixture for each urine sample according to our recently established automated method 26. Briefly, 6 mg Oasis MAX resins and 20 mg C18 resins were stacked into tips to generate the mix-mode enrichment tip. The tips were sequentially conditioned by 100% ACN, 100 mM Triethylammonium Acetate (TAAB), 95% ACN containing 1% TFA, and 0.1% TFA (20 cycles each). Peptide mixtures from urine samples were dissolved in 0.1% TFA and put in 96-well plate, then loaded onto MAX/C18 tips with 15 cycles of aspirating/dispensing followed by a rinse with 0.1% TFA (10 cycles). Peptides were desalted via binding onto C18. Desalted intact glycopeptides were eluted from C18 to MAX using 95% ACN / 1% TFA. Finally, the bound intact glycopeptides were eluted from MAX by 50% ACN / 0.1% TFA and dried down. For the removal of N-glycans, intact glycopeptides were dissolved in 100 mM Tris-HCl at pH 8.0 with 2 μL of PNGase F. The mixture was incubated in 37 °C overnight and subjected to C18-cleanup via StageTip method 28. After removing N-glycans, N-linked glycosite-containing peptides (one tenth of the total glycopeptides enriched from 500 µL urine) were subjected to DIA MS analysis together with index retention time (iRT) peptides in a Q-Exactive HF-X mass spectrometer.

### Basic reversed-phase liquid chromatography (bRPLC) fractionation

To build a PCa urine specific spectral library for direct database searching of the DIA data, glycopeptides from 142 human urine samples (discovery cohort) were pooled and fractionated by bRPLC for a deeper coverage of low abundance peptides. The pooled glycopeptides were load onto reversed-phase Zorbax Extend-C18 analytical column (1.8 μm resin, 4.6×100 mm, Agilent Technology, CA), which was installed on an Agilent 1220 Infinity HPLC system. With buffer A (10 mM ammonium formate, pH 10) and buffer B (10 mM ammonium formate in 90% ACN, pH 10), the HPLC gradient was set as follows: 0-2% B for 10 min followed by 2-8% B for 5 min, 8-35% B for 85 min, 35-95% B for 5 min, and 95-95% B for 15 min. Total of 96 fractions were collected in a time-based mode from 16 to 112 min and were concatenated into 24 fractions. The 24 fractions were further consolidated into eight final fractions. The final pooled fractions were dried down and then dissolved in 0.1% FA together with iRT peptides for data-dependent acquisition (DDA) analysis.

### LC-MS/MS analysis of glycopeptides

For the DDA MS analysis, all samples were analyzed by a Q-Exactive HF-X mass spectrometer connected to an EASY-nLC 1200 system (Thermo Fisher Scientific). Glycopeptides were directly injected into a 28 cm long self-packed C18 column (1.9 μm/120 Å ReproSil-Pur C18 resin, Dr. Maisch GmbH, Germany) with an integrated PicoFrit emitter (New Objective). Peptides were separated using 88 min gradient from 5% to 40% buffer B (80% ACN and 0.1% formic acid) at a flow rate of 300 nL/min. MS1 was acquired at a resolution of 60,000 from m/z 400 to 1000 with automatic gain control (AGC) set at 1×10^6^ and a max injection time of 60 ms. MS2 scans were performed by higher-energy collisional dissociation (HCD) on the top 20 abundant precursor ions at a resolution of 15,000 with an isolation width of 1.4 m/z and a normalized collision energy (NCE) of 30. The dynamic exclusion was set as 20 s.

DIA MS analysis was performed on the same MS instrument and LC separation gradient was kept consistent with the DDA analysis. The setting of full MS scan was similar as the MS1 scan of DDA, except the resolution was 120,000 under DIA mode. For the DIA MS2 scan, a set of 50 overlapping windows was constructed covering the precursor mass range of 400-1000 Da with a fixed isolation width of 12 m/z. The resolution and AGC was the same as that of a full MS scan with a maximum injection time of 25 ms and NCE of 30.

### Construction of a PCa urine specific spectral library using DDA data

For generation of the PCa urine-specific spectral library, glycopeptides enriched from the 142 urine samples (discovery cohort) were pooled together and measured in two ways. The unfractionated samples were analyzed with DDA in three technical replicates. The eight fractions generated by the bRPLC fractionation method were also measured by DDA MS. In addition, glycopeptides enriched from one urine specimen (sample name: P2) were also subjected to DDA analysis. The 12 DDA raw files were searched against a combined database consisting of an iRT fusion protein and human proteins (Swiss-Prot, downloaded on 02/20/2019) via Pulsar algorithm embedded in Spectronaut Pulsar X (Biognosys, Zurich, Switzerland). The parameters for the database search are as follows: an allowance for tryptic peptides of up to two missed cleavages within the length range of 2 to 52 amino acids. Mass tolerance of MS1 and MS2 were set as dynamic with a correction factor of one. Carbamidomethylation of cysteine (C) was set as a fixed modification whereas oxidation of methionine (M) and acetylation of protein N-terminal were selected as variable modifications. Since N-glycosylated asparagine (N) is converted to aspartic acid (D) upon PNGase F treatment, conversion of N to D was set as a variable modification as well. A false discovery rate (FDR) of < 1% was required to generate the final peptide spectral library, in which there were 1289 unique glycopeptides of 594 glycoproteins.

### Database search and statistical analysis of glycopeptide DIA data

For quantitative analysis of glycopeptides across the urine samples, DIA raw data files were first searched against the aforementioned spectral library for identification of glycopeptides followed by the quantification via Spectronaut Pulsar X. Mass tolerance of MS and MS/MS was set as dynamic with a correction factor of one. Source-specific iRT calibration was enabled with a local (non-linear) RT regression. Cross run normalization was not selected. All quantified glycopeptides were filtered by a Q value cutoff of 0.01 (which corresponds to an FDR of 1%) and decoy peptide sequences were removed.

We performed normalization on glycopeptides to the total protein amount in individual urine sample then multiplied by the median of the total amount of proteins across samples. We used WebGestalt for Gene Ontology (GO) cellular component annotation 29. Enriched pathways were analyzed using STRING 30. At the initial discovery phase, glycopeptides identified and quantified in at least one-third of AG or NAG samples were selected. The p-value for each glycopeptide in the discovery cohort was computed between AG and NAG groups using the Mann-Whitney U test and multiple testing by label permutation was used to estimate the false discovery rate (FDR) of candidate marker selection. For each glycopeptide, its discrimination power as an individual marker or in combination with serum PSA through logistic regression was evaluated using receiver operating characteristic (ROC) analysis in three repeated 5-fold cross validations. The mean ROC curves from repeated 5-fold cross validation were depicted and area under the curve (AUC) was computed for the mean ROC curves. The predictive models with cross validation were built using caret (version 6.0-85) 31 in R. ROC curves were generated using pROC (version 1.13). AUC along with 95% confidence interval (95% CI) as well as sensitivity and specificity at the best cutoff point along with 95% CI were obtained via MLeval 32 in R, for which the best cutoff point on the ROC curve has the maximal summed sensitivity and specificity. The generated predictive models were further investigated using another validation cohort.

## Results and Discussion

### Workflow of an integrated urine glycoproteomic analysis

**Figure 1:**
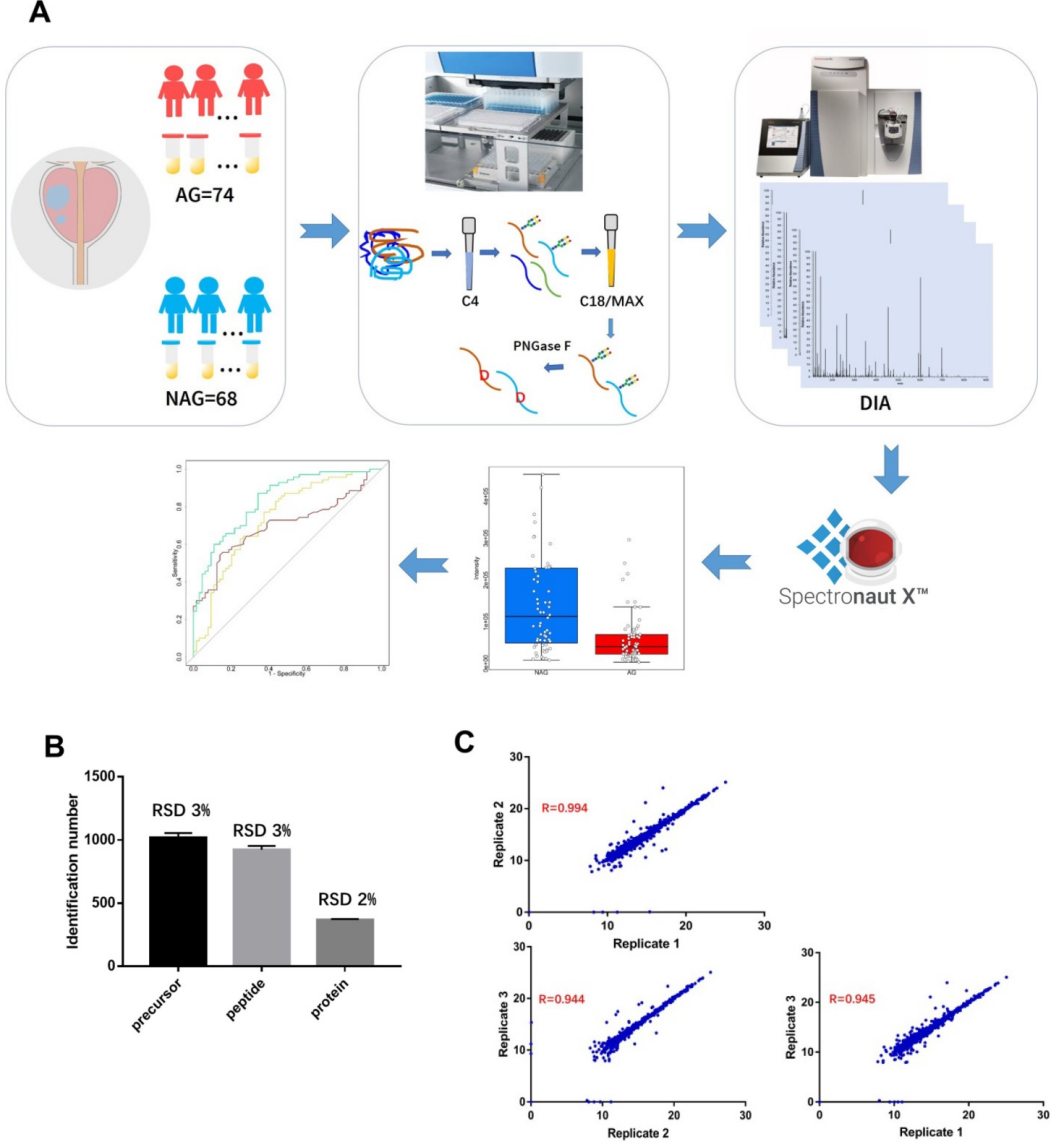
A. Experimental workflow for the quantitative analysis of urine glycoproteomic to discover candidate biomarkers associated with aggressive prostate cancer. Reproducibility of DIA MS analysis was shown. B. The relative standard deviation (RSD) of the identification number of peptide precursors, peptides and proteins over three replicate DIA runs of glycopeptides are less than 3%. C. The correlation coefficients between any two replicates was at least 0.944.

It is essential to establish an experimental workflow that can analyze a large number of specimens with high throughput, high sensitivity, and high reproducibility for biomarker discovery using urine. A previous study demonstrated the feasibility of detecting a glycoprotein difference between aggressive PCa and non-aggressive PCa using pooled urine samples from PCa patients 24. However, the performance of each glycoprotein for differentiating aggressive and non-aggressive PCa was difficult to evaluate using pooled urine samples. Therefore, in this study, we established an integrated workflow by coupling automated urine sample preparation with DIA MS for the quantitative analysis of N-linked glycosite-containing peptides (referred to as De-N-glycopeptides or glycopeptides for simplicity) derived from the urine samples of 74 AG (Gleason score ≥8) and 68 NAG (Gleason score = 6) PCa patients (discovery cohort, Table S1). An overview of the experimental workflow is illustrated in Figure 1A. Table S2 shows the major differences between the previous study 24 and the current study in terms of samples, the glycopeptide enrichment method, data acquisition, and quantification approach.

To perform the quantitative proteomic analysis of the enriched glycopeptides using DIA, a PCa urine specific spectral library was generated using the glycopeptides from the discovery cohort via DDA MS (Table S3). The constructed spectral library contained 1,289 unique de-N-glycopeptides corresponding to 594 glycoproteins, thereby allowing for a broad coverage of the PCa-related urine glycoproteome for reliable DIA data analysis. Quantification accuracy and data reproducibility are extremely important for biomarker discovery. Thus, the reproducibility of DIA MS was evaluated by using three replicate injections. The relative standard deviation (RSD) of the identification number of peptide precursors (*i.e.*, the same peptide sequences with different charge states or modifications), peptides, or proteins across the three replicates was 3% or less, which indicates consistency in DIA MS data acquisition (Figure 1B). To determine reproducibility among replicates, pair-wise correlation was calculated based on the intensity of quantified peptide precursors (Figure 1C). The correlation between any two replicates was ≥0.944 indicating the precision in quantification of our DIA MS method.

To investigate the levels of non-enzymatic deamidation generated during sample preparation and assess the false discovery rate of N-linked glycopeptides caused by non-enzymatic deamidation, a control experiment was performed in 20 randomly selected urine specimens from the discovery cohort. The urine proteins were subjected to trypsin digestion followed by enrichment of intact glycopeptides. The enriched intact glycopeptides from each sample were divided into two equal aliquots. One aliquot was directly analyzed by LC-MS/MS without PNGase F treatment. For the other aliquot, peptides were first treated with PNGase F to remove glycans before LC-MS/MS analysis. The identified peptides from the 20 urine specimens are presented in Table S4. For the 20 samples without PNGase F treatment, 2132 peptides were identified in total, of which 109 peptides (5.1%) were modified by deamidation and only 5 out of 109 deamidated peptides (4.6%) contained the NXS/T sequence. The result indicates that the identification rate of false N-linked glycopeptides (generated by nonenzymatic deamidation in NXS/T motif) is low (0.2%)). For the PNGase F treated peptides from the same 20 urine samples, 2692 peptides were identified, in which 1652 peptides (61.4%) were modified by deamidation (Table S4). Among the 1652 peptides modified by deamidation, 1458 (88%) of them contained an NXS/T motif in their peptide sequences. Therefore, we can conclude that most of the deamidated peptides, particularly the deamidated peptides with NXS/T motif, were generated due to removal of glycans using PNGase F.

### Overview of the quantified glycopeptides in the discovery cohort

For the DIA MS analysis of each clinical urine sample, glycopeptides enriched from the 142 samples (discovery cohort) were analyzed. In total, 889 glycopeptides originating from 549 glycoproteins were identified and quantified in this study at an FDR of <1% for both proteins and peptides (Table S5). We further investigated the cellular component of the identified proteins based on the GO annotation 33. Consistent with previous reports 9, 34, a majority of glycopeptides identified from urine were proteins derived from membrane (373 glycopeptides, 67.9%), extracellular space (312 glycopeptides, 56.8%), or otherwise secreted (304 glycopeptides, 55.4%), indicating that most of the glycopeptides were originated from glycoproteins secreted or shed from tissues. For the biological pathway analysis, the most significantly enriched pathways were neutrophil degranulation, innate immune system, immune system, and extracellular matrix organization.

### Quantitative analysis of the urinary glycoproteins

**Figure 2:**
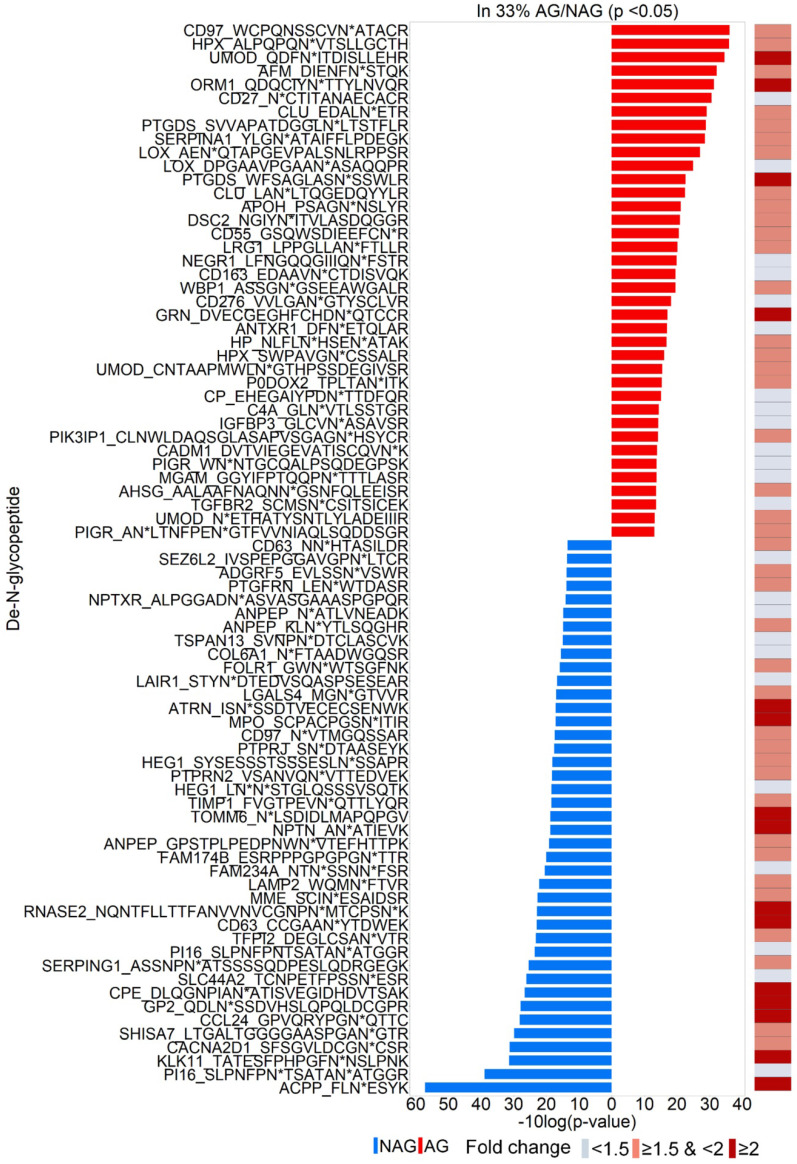
Identifications of 79 glycopeptides with significant fold change between AG and NAG samples (*p*<0.05). Glycopeptides with elevated levels in AG samples and NAG samples are in red and blue, respectively. The right panel shows the fold change of the glycopeptides between AG and NAG samples.

To discover urinary glycoproteins associated with aggressive PCa, a two-tier screening approach of candidate selection was used to narrow down our initial targets. The first-tier retained glycopeptides quantified in at least one-third of the AG or NAG samples. The second-tier was to filter further and keep only those significantly changed between AG and NAG samples with *p* < 0.05. In total, 79 glycopeptides were identified (Table S6), where 38 increased and 41 decreased in AG group relative to NAG group (Figure 2), were selected for further evaluation. Among the 79 glycopeptides with significant changes, 54 glycopeptides had at least a 1.5-fold change between AG and NAG groups (Table S7 and the right panel of Figure 2) with an estimated FDR of 0.25 based on label permutation.

### Determining the utilities of urinary glycoproteins for the detection of aggressive PCa

To evaluate the discrimination power of the differentially expressed glycoproteins in distinguishing AG PCa from NAG PCa, the ROC curves were generated and AUC were calculated based on predictive models of logistic regression with three repeated 5-fold cross validation for the 54 glycopeptides, where 29 showed decreased levels (Table S7) and 25 showed increased levels (Table S7) in AG PCa relative to NAG PCa. From the ROC results, a total of 20 candidates were selected of which 9 were decreased and 11 were increased in AG PCa samples compared to NAG PCa samples (Table S8 with blue highlights). In this study, serum PSA concentration obtained from clinical testing served as a reference to determine if candidates were comparable to serum PSA result or could be used in combination with the serum PSA to further improve the discrimination power towards AG PCa. Detailed information on the selected 20 candidates is in Table S9.

Among the 29 glycopeptides showing lower expression in AG PCa, we found glycopeptide FLN*ESYK from ACPP (Prostatic acid phosphatase, * indicates the glycosylation site) showed the best performance. ACPP is a prostate specific protein with at least fifty-fold higher mRNA expression levels in prostate tissue compared to other tissues 35. Moreover, decreased expression level of ACPP has been found in the tumor tissues of aggressive PCa patients compared to non-aggressive PCa patients based on quantitative glycoproteomic study 23 and immunohistochemistry analysis of ACPP on the cancer slides of PCa patients 36. ACPP acts as a tumor suppressor of PCa through dephosphorylation of ERBB2 (receptor tyrosine-protein kinase erbB-2) and deactivation of MAPK-mediated (mitogen-activated protein kinase) signaling 37, 38. Decreased ACPP expression correlates with the activation of downstream MAPK signaling resulting in PCa progression as well as androgen independent growth of PCa cells 37, 38.

**Figure 3:**
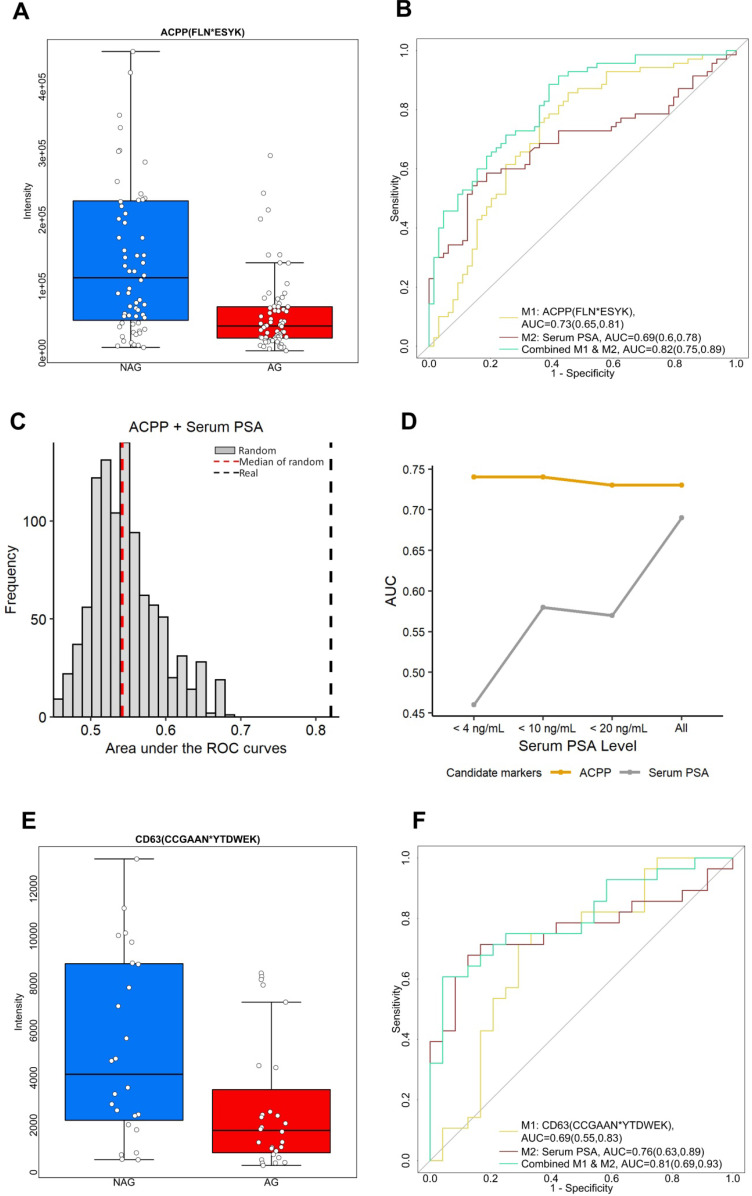
Two down-regulated glycopeptides in AG PCa. A. Expression profiles of urinary ACPP (FLN*ESYK) in AG PCa and NAG PCa samples. B. ROC analysis results of urinary ACPP and serum PSA. C. A panel comprising urinary glycopeptide from ACPP and serum PSA was evaluated by label permutation for 1000 times. The AUC distribution of the 1000 random models (median AUC=0.55, red dotted line) was compared to the real model (AUC=0.82, black dotted line). D. Effect of serum PSA concentrations on the performance of urinary ACPP for detecting AG PCa. The AUC of urinary ACPP and serum PSA for detecting AG PCa was calculated and compared at different serum PSA cutoffs. E. Expression profiles of CD63 (CCGAAN*YTDWEK) in AG PCa and NAG PCa urine samples. F. ROC analysis results of CD63 (CCGAAN*YTDWEK) and serum PSA. The boxplots display a summary of minimum, first quartile, median, third quartile, and maximum of the expression profiles for AG and NAG PCa samples. AUC and 95% confidence interval are depicted for each candidate marker.

ACPP was initially discovered as a serum biomarker for PCa instead of a urinary biomarker. Serum ACPP was measured by its elevated activity in the patient with PCa rather than by protein abundance 38, 39. However, the assays to measure the activity of the serum ACPP were unstable in room temperature causing technical variations 40, 41. Consequently, when serum PSA emerged and demonstrated better accuracy in detecting PCa 42, serum ACPP soon fell to disfavor. In the current study, we discovered the prognostic value of glycopeptide from urinary ACPP. Glycopeptide FLN*ESYK of ACPP was identified in 70 of AG and 64 of NAG urine samples, with intensity significantly decreased in AG PCa group (fold change=2.56 with *p* < 0.01, Table S9 and Figure 3A). As shown in Figure 3B, urinary ACPP had a better predictive power with AUC of 0.73 (95% CI, 0.65 to 0.81) compared to serum PSA with AUC of 0.69 (95% CI, 0.6 to 0.78) (Table S8 and Figure 3B). Since ACPP (low in AG PCa samples, where median of AG=40547.17 and median of NAG=113063.9) and serum PSA (high in AG PCa samples, where median of AG=7.8 and median of NAG=4.5) had opposite expression profiles with a negative Spearman's correlation of -0.023 suggesting that they may provide complementary information for AG PCa diagnosis. Therefore, a two-signature panel consisting of ACPP and serum PSA was examined. We found the panel provided better diagnostic accuracy by improving the AUC to 0.82 (95% CI, 0.75 to 0.89) (Figure 3B and Table S8). To ensure the performance of the panel (urinary ACPP + serum PSA), we generated 1000 random combined signature sets using label permutation and computed the AUC for each built random model (Figure 3C). The random models generated a median AUC of 0.55, which was lower and clearly separated from our real model (AUC=0.82).

To investigate the effect of serum PSA concentration on the performance of urinary ACPP in detecting AG PCa, ROC analysis was conducted for urinary ACPP and serum PSA at different serum PSA cutoffs. As shown in Figure 3D, the performance of urinary ACPP is quite consistent across different cutoff points with the AUC ranged from 0.73 to 0.74. On the contrary, the performance of serum PSA varied at different cutoff values with the AUC ranged from 0.45 to 0.69. It has demonstrated limited discrimination power of serum PSA towards AG PCa detection with serum PSA values < 20 ng/mL (Figure 3D). Although further investigation is required, the aforementioned result indicates that the performance of urinary ACPP is independent of serum PSA concentrations. Thus, urinary ACPP may be useful in supplementing serum PSA test for the detection of AG PCa at serum PSA ranges less than 20 ng/mL.

Another down-regulated candidate glycopeptide of interest is CD63 (CD63 antigen). CD63 is one of the widely accepted exosomal markers that belongs to the transmembrane 4 superfamily (TM4SF) 43. Protein complexes formed by TM4SF members are associated with beta-1 integrin and contribute to cell motility, which plays an important role in tumor progression 43, 44. In our study, glycopeptide (CCGAAN*YTDWEK) from CD63 was identified and its intensity was 2.2 times lower in AG PCa urine specimens comparing to NAG PCa urine specimens (*p* < 0.05, Figure 3E). Figure 3F shows that the glycopeptide has the ability to differentiate AG PCa from NAG PCa with an AUC of 0.69 (95% CI, 0.55 to 0.83). When combined with serum PSA, the AUC was further improved (0.81, 95% CI of 0.69 to 0.93, Table S8).

Besides ACPP and CD63, glycopeptides from other proteins such as ATRN (Attractin), GP2 (Pancreatic secretory granule membrane major glycoprotein GP2), KLK11 (Kallikrein-11), PTPRN2 (Receptor-type tyrosine-protein phosphatase N2), NPTN (Neuroplastin), CPE (Carboxypeptidase E), and RNASE2 (Non-secretory ribonuclease), also showed good performance in detecting AG PCa when combined with serum PSA (Table S8). In addition, TMPRSS2 (Transmembrane Serine Protease 2) is another important PCa-specific protein identified in this study. TMPRSS2 is an androgen-responsive gene. Its fusion to ERG contributes to the development of androgen-independence in PCa, resulting in cancer progression including invasion and metastasis 45. Genetic detection of this type of fusion in urine specimens has entered clinical practice 46-48. In this study, we found the expression levels of LN*TSAGNVDIYK from TMPRSS2 was 1.7-fold decreased in AG PCa urine samples (n=25) relative to NAG PCa urine samples (n=22) with a p-value of 0.13. Usually, the exon 1 or 2 of TMPRSS2 is fused to exon 2 or 4 of ERG during TMPRSS2:ERG fusion 45. However, the glycosite, N213, on LN*TSAGNVDIYK is located after the fusion position. We speculate that the TMPRSS2:ERG fusion may lead to the decrease of LN*TSAGNVDIYK expression. Therefore, a decrease in the level of LN*TSAGNVDIYK in AG PCa urine samples may be explained by TMPRSS2:ERG fusion more frequently occurred in AG PCa samples. Further studies are needed to investigate this hypothesis. Nonetheless, our finding may provide a new angle for studying the TMPRSS2:ERG fusion.

**Figure 4:**
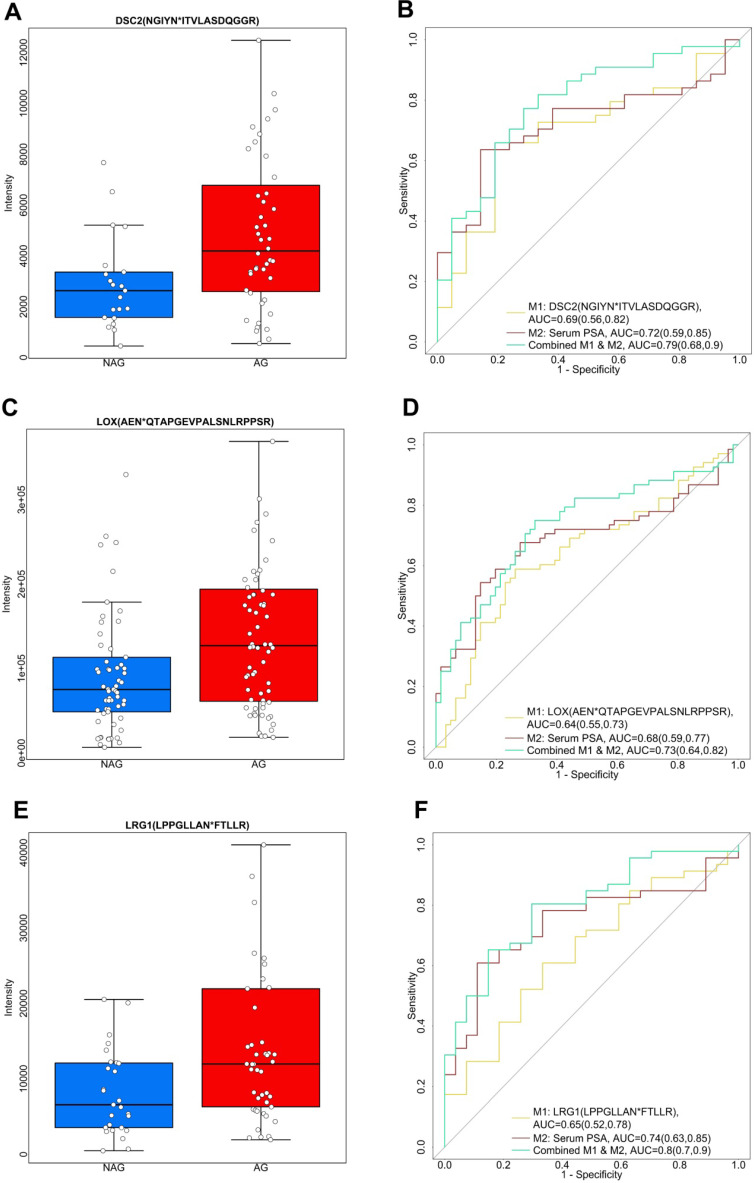
Three up-regulated glycopeptides in AG PCa. A. Expression profiles of DSC2 (NGIYN*ITVLASDQGGR) in AG and NAG PCa urine samples. B. ROC analysis results of DSC2 (NGIYN*ITVLASDQGGR) and serum PSA. C. Expression profiles of LOX (AEN*QTAPGEVPALSNLRPPSR) in AG PCa and NAG PCa urine samples. D. ROC analysis results of LOX (AEN*QTAPGEVPALSNLRPPSR) and serum PSA. E. Expression profiles of LRG1 (LPPGLLAN*FTLLR) in AG PCa and NAG PCa urine samples. F. ROC analysis results of LRG1 (LPPGLLAN*FTLLR) and serum PSA. The boxplots display a summary of minimum, first quartile, median, third quartile, and maximum of the expression profiles for AG and NAG PCa samples. AUC and 95% confidence interval are depicted for each candidate marker.

Apart from the aforementioned down-regulated glycopeptides, we also explored the up-regulated candidate glycopeptides including NGIYN*ITVLASDQGGR from DSC2 (Desmocollin-2), AEN*QTAPGEVPALSNLRPPSR from LOX (Protein-lysine 6-oxidase), and LPPGLLAN*FTLLR from LRG1 (Leucine-rich alpha-2-glycoprotein), as shown in Figure 4. DSC2 belongs to the demecolcine protein subfamily and is the major component of desmosomes. Desmosomes are involved in establishing and maintaining cell-cell adhesion and are critical for the development, differentiation, and maintenance of normal human tissues 49. The loss of cell-cell adhesion is frequently associated with the progression of PCa to a metastatic state. Previous research has found aberrant expression of DCS2 in several types of cancers by possible involvement in tumor progression 50. In our study, we observed an elevated expression profile of the glycopeptide, NGIYN*ITVLASDQGGR of DSC2, in AG PCa urine samples (Figure 4A). NGIYN*ITVLASDQGGR of DSC2 generated an AUC of 0.69 (95% CI, 0.56 to 0.82) as an individual signature. However, an improvement was noticed when combined with serum PSA with an AUC of 0.79 (95% CI, 0.68 to 0.9) (Figure 4B and Table S8). LOX is another glycopeptide of interest since it is reported to be associated with PCa 51. We observed the level of AEN*QTAPGEVPALSNLRPPSR from LOX was higher in AG PCa compared to NAG PCa (Figure 4C). The ROC analysis included AEN*QTAPGEVPALSNLRPPSR of LOX and serum PSA indicating that the combination of the two enhanced the separation of AG PCa from NAG PCa (AUC of 0.73, 95% CI of 0.64 to 0.82) in comparison with using LOX (AUC of 0.64, 95% CI of 0.55 to 0.73) and serum PSA (AUC of 0.68, 95% CI of 0.59 to 0.77) individually (Figure 4D and Table S8). Furthermore, LRG1 (Leucine-rich-alpha-2-glycoprotein-1), an inflammatory protein in human serum participates in the immune response 52, was identified as an up-regulated glycopeptide in this study. LRG1 was previously recognized as a new oncogene-associated protein promoting dysfunctional vessel growth 53. The up-regulation of LRG1 has been reported to be associated with progression and angiogenesis of multiple cancers 54-56. Here, LPPGLLAN*FTLLR from LRG1 was expressed twice more in AG PCa than NAG PCa urine samples (Figure 4E). A combined use of LRG1 and serum PSA generated an AUC of 0.8 (95% CI, 0.7 to 0.9) (Figure 4F and Table S8) suggesting the potential of LRG1 as a urinary glycoprotein for aggressive PCa. In addition to DSC2, LOX and LRG1, glycopeptides from other glycoproteins including CLU (Clusterin), SERPINA1 (Alpha-1-antitrypsin), ORM1 (Alpha-1-acid glycoprotein 1), PTGDS (Prostaglandin-H2 D-isomerase), GRN (Progranulin), UMOD (Uromodulin), AFM (Afamin) and CD97 (CD97 antigen), were also found to be significantly (*p* < 0.05) elevated in AG PCa urine specimens and the capacity in group separation was evaluated by ROC analysis (Table S8).

### The combined performance of two urinary glycoproteins and serum PSA for the detection of AG PCa

**Figure 5:**
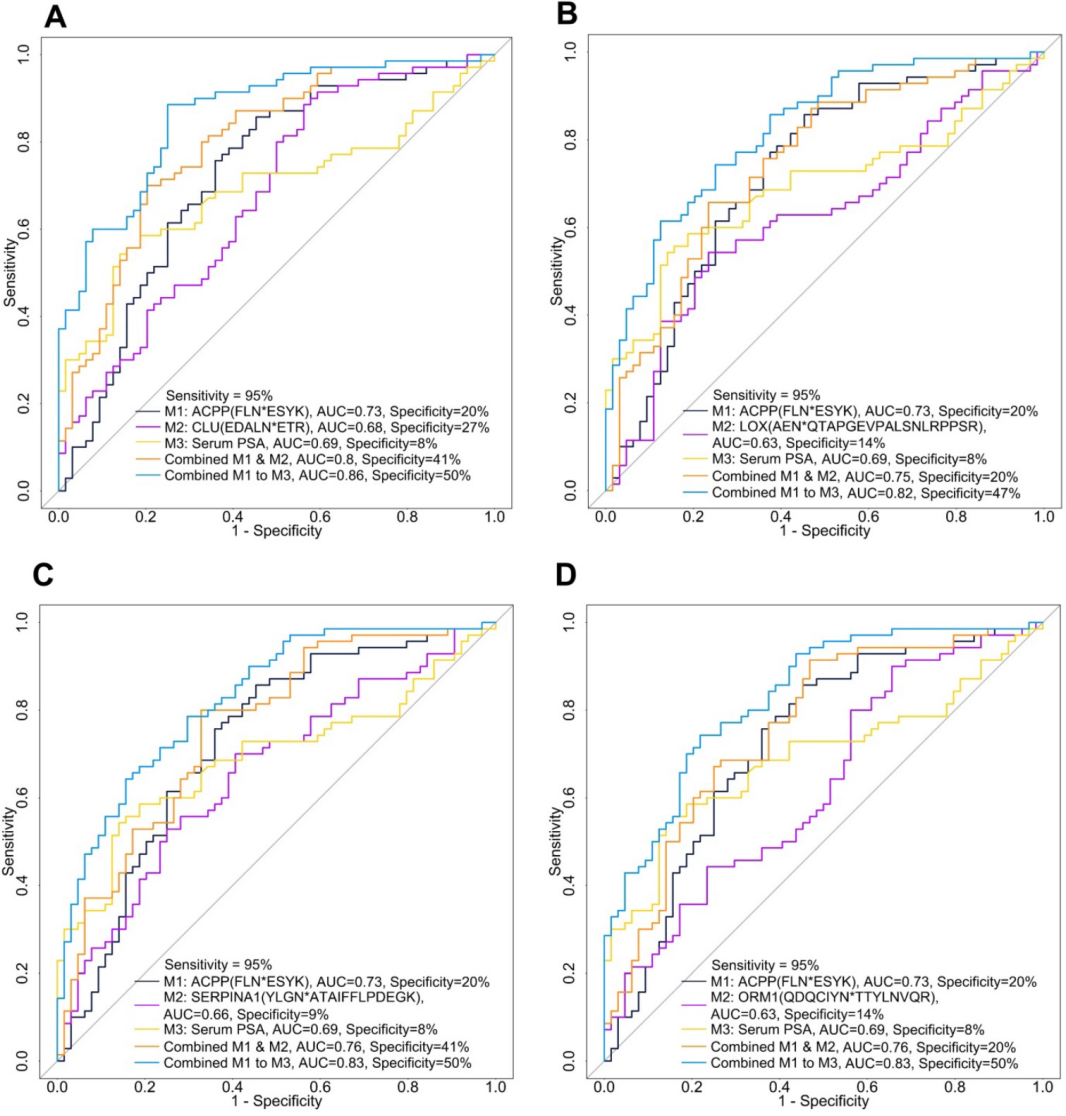
ROC analysis of combined panels including urinary ACPP (FLN*ESYK), one up-regulated glycopeptide, and serum PSA. A. The combinatory performance of ACPP (FLN*ESYK), CLU (EDALN*ETR) and serum PSA. B. The combinatory performance of ACPP (FLN*ESYK), LOX (AEN*QTAPGEVPALSNLRPPSR) and serum PSA. C. The combinatory performance of ACPP (FLN*ESYK), SERPINA1 (YLGN*ATAIFFLPDEGK) and serum PSA. D. The combinatory performance of ACPP (FLN*ESYK), ORM1 (QDQCIYN*TTYLNVQR) and serum PSA.

After evaluating the glycopeptide signatures individually and in combination with serum PSA, we further investigated the potential of combining a down-regulated glycopeptide and an up-regulated glycopeptide since this may improve the clinical utility of these candidate glycopeptides. We selected urinary ACPP as the primary down-regulated candidate glycopeptide because it had the best performance among the candidate glycopeptides. To directly compare the performance of urinary ACPP with different up-regulated candidate glycopeptides, we fixed the sensitivity at 95% and then compared the specificity. By setting a very high sensitivity, even at the cost of reduced specificity, would also fulfill the need in clinical practice for lowering misdiagnosis of patients with aggressive PCa. The ROC results of different panels are shown in Table S10, where the four panels demonstrated relatively better performance in distinguishing AG PCa from NAG PCa (higher AUC and higher specificity at 95% sensitivity) are presented in Figure 5.

Among all the two-signature panels (*i.e.*, the combination of urinary ACPP and an up-regulated glycopeptide), urinary ACPP combined with urinary CLU had the best performance (AUC=0.8) achieving a specificity of 41% at 95% sensitivity (Figure 5A and Table S10). By adding serum PSA into the panel, the AUC was improved to 0.86 and specificity was increased to 50% at 95% sensitivity (Figure 5A and Table S10). While serum PSA itself generated an AUC of 0.69, and the specificity was only 8% at 95% sensitivity. Since ACPP and CLU were detected in more than 91% of the samples, further clinical use of the combined signature panel is possible. The combination of urinary ACPP, urinary LOX, and serum PSA as a three-signature panel also displayed a good capacity in differentiating AG from NAG PCa (AUC=0.82, Figure 5B and Table S10). Furthermore, urinary ACPP combined with urinary SERPINA1 (Figure 5C and Table S10) and urinary ACPP combined with urinary ORM1 (Figure 5D and Table S10) both achieved an AUC of 0.76. Additional improvement in discrimination power was observed when serum PSA was included; the AUC increased to 0.83 with specificity reached to 50% at 95% sensitivity (Figures 5C-D). These results demonstrate that a combined signature panel composed of one up, one down-regulated glycopeptides from urinary glycoproteins, and serum PSA has the ability to distinguish AG and NAG PCs patients, where the two glycopeptide signatures serve as adjuncts to serum PSA test to gain improved discrimination power. In conclusion, three-signature panels discovered using quantitative glycoproteomic strategy showed better performance than individual signatures for the detection of AG PCa.

### Validation of the glycopeptide signatures using a validation cohort

**Figure 6:**
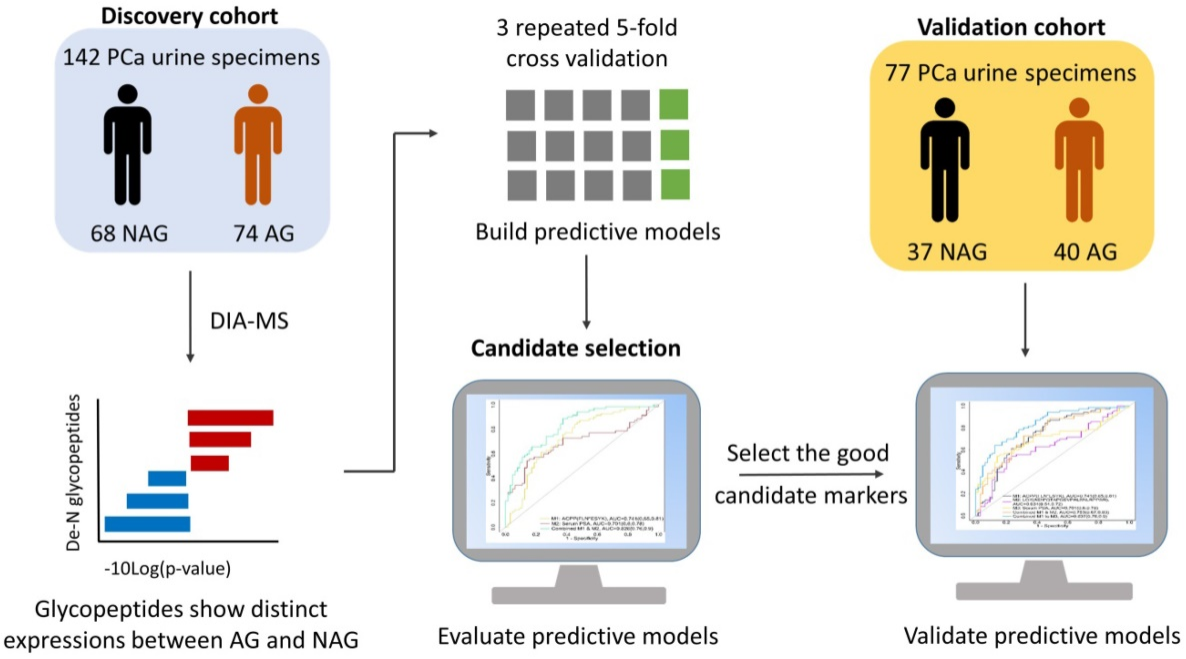
Schematic overview of candidate glycopeptide discovery and validation.

A validation cohort composed of 40 AG and 37 NAG PCa patients was analyzed to further assess the performance of the identified glycopeptides and the predictive models from the discovery cohort (Figure 6). Among the 20 candidate glycopeptides selected based on ROC analysis in discovery set (Table S9), 13 of them showed the same trend in their expression profiles as they did in discovery cohort (Table S11).

Next, we evaluated the performance of the candidate glycopeptides that can distinguish AG from NAG PCa during the discovery phase, including glycopeptides from ACPP, CD63, DSC2, LOX and LRG1 (Figures 3 and 4), using the validation cohort. Among them, the predictive power of glycopeptides from DSC2 and LRG1 decreased in the validation cohort (Table S12), suggesting that further rigorous validation is needed to evaluate their potential diagnosis utility for aggressive PCa. Nevertheless, ACPP, CD63 and LOX performed consistently between the discovery and validation cohorts, either as individual biomarkers or in combination with serum PSA (Table S12). For example, ACPP remained as a reliable candidate biomarker for distinguishing AG from NAG PCa, especially when combined with serum PSA to achieve an AUC of 0.83 (95% CI of 0.74 to 0.83) in the validation cohort (Table 1 and Table S12), which was comparable to that of the discovery cohort (AUC=0.82, 95% CI of 0.75 to 0.89).

We further evaluated the performances of the predictive models composed of three candidate biomarkers. Combining ACPP and serum PSA with one of the elevated urinary signatures (CLU, LOX, ORM1, or SERPINA1) showed good performances relative to the other panels for detecting AG PCa in the discovery set (Table S10 and Figure 5). The four panels were successfully validated and consistent performances were observed between the discovery and validation cohorts (Table S13 and Table 1). Taking the three-signature panel consisting of urinary ACPP, urinary LOX, and serum PSA as an example, the AUCs were 0.82 and 0.85 in the discovery cohort and the validation cohort, respectively, indicating the stable performance of the predictive model of this panel.

However, we later found that 24 NAG urine samples in the validation sample cohort were collected from a subset of patients in the discovery cohort. Since the entire sample preparation and DIA-MS analysis processes were carried out independently for the discovery and validation cohorts, we still present the evaluation results using the entire validation cohort (referred to as validation set 1). By removing the overlapped patient samples, a subset of the validation set consisting of 40 AG and 13 NAG urine samples was used (referred to as validation set 2) to conduct another evaluation (Table 1 and Table S11-13). As shown in Table 1, the discrimination power of ACPP combined with serum PSA was slightly lower using validation set 2 (AUC= 0.8), which was possibly related to the smaller sample size of the second validation set. An AUC of 0.81 was found for the three-signature panel consisting of urinary ACPP, urinary LOX, and serum PSA in validation set 2 (Table 1 and Table S13). A similar outcome was observed for the five individual candidate biomarkers comparing validation set 1 to validation set 2 (Table S12). Collectively, novel panels of candidate biomarkers for aggressive PCa were discovered, and they performed consistently in an independent validation cohort. For clinical applications of the biomarker panels, the next phase of our study would be to validate the panels using larger urine sample cohorts from multi-centers to further assess their reliability.

## Conclusion

Despite the prevalence of PCa, there is still a lack of biomarkers for identifying aggressive PCa. Therefore, developing a noninvasive test for the early detection of aggressive PCa is necessary. The aim of this study is to detect glycopeptides of urinary glycoproteins associated with aggressive PCa. By applying a high throughput and integrated workflow involving automation in the urine sample preparation, DIA MS, and quantitative analysis of the urine glycoproteome, we were able to evaluate the performance of glycopeptides from the 142 urine samples (discovery cohort) for the aim of detecting aggressive PCa.

Based on our analysis, 79 glycopeptides were significantly altered between AG and NAG samples (*p* < 0.05), 54 of which having at least a 1.5-fold change. Moreover, 20 glycopeptides were identified as candidates associated with aggressiveness in PCa. Glycopeptide FLN*ESYK from ACPP showed the best performance as an individual candidate biomarker compared to other candidates; further improvement was observed when combined with the traditional serum PSA. In addition, the performance of urinary ACPP is independent of serum PSA concentrations; thus, it can serve as an adjunct to serum PSA for the detection of aggressive PCa particularly for patients with lower level of serum PSA. Glycopeptides from CD63 and LOX also showed potential as noninvasive urinary glycoproteomic biomarker for aggressive PCa with consistent performances across the discovery and validation cohorts. Notably, the four three-signature panels comprising of urinary ACPP; urinary CLU, LOX, ORM1, or SERPINA1; and serum PSA outperformed individual signatures when it came to detecting AG PCa. The three-signature panel composed of ACPP, CLU and serum PSA can discriminate aggressive PCa at an AUC of 0.86. The predictive models with good performance were further investigated using a validation cohort. Consistent results were found between the discovery and validation cohorts, indicating the reliability of the candidates.

Our study highlights the application of a high-throughput and highly reproducible automated urine glycopeptide preparation platform coupled with DIA MS for the discovery of glycoproteins associated with aggressive PCa. While the novel panels of multiple signatures discovered in this study demonstrate the potential of characterizing and detecting the aggressiveness of PCa, substantial work is still needed before they can be used for clinical applications. The next phase of our research will include the following: (1) Validate the urinary candidate biomarkers using a large-scale cohort. (2) Perform multi-center validation. (3) Make systematic comparisons of the glycoproteomic candidate biomarkers found in this study with other urinary biomarkers for PCa (e.g. urinary RNA biomarker PCA3) to investigate whether they can supplement each other to generate a new panel of noninvasive urinary biomarkers with improved discrimination power. Furthermore, aberrant glycosylation has been recognized as a hallmark in oncogenic transformation and plays an important role in cancer development and progression. Thus, studying glycosylation patterns will help in biomarker discovery. For instance, fucosylated PSA displays a better predictive power to differentiate aggressive from non-aggressive PCa 57, 58. Therefore, we will also dedicate our efforts to investigate the glycosylation forms of our glycoproteins in order to further improve the diagnostic accuracy of aggressive PCa.

## Supplementary Material

Supplementary tables.

## Figures and Tables

**Table 1:** Performance of different panel of candidate biomarkers in discovery cohort (74 AG and 68 NAG), validation cohort (set 1: 40 AG and 37 NAG; set 2: 40 AG and 13 NAG)

Panel of candidate biomarkers	Area under the ROC curves (95% confidence interval)		
	Discovery cohort	Validation cohort	
		40 AG and 37 NAG (set 1)	40 AG and 13 NAG (set 2)
ACPP & Serum PSA	0.82 (0.75,0.89)	0.83 (0.74,0.92)	0.8 (0.67,0.93)
ACPP & CLU & Serum PSA	0.86 (0.8,0.92)	0.85 (0.76,0.94)	0.76 (0.6,0.92)
ACPP & LOX & Serum PSA	0.82 (0.75,0.89)	0.85 (0.76,0.93)	0.81 (0.69,0.93)
ACPP & SERPINA1 & Serum PSA	0.83 (0.76,0.9)	0.84 (0.75,0.93)	0.82 (0.7,0.94)
ACPP & ORM1 & Serum PSA	0.83 (0.76,0.9)	0.82 (0.72,0.91)	0.82 (0.71,0.94)

## Abbreviations

Afamin: AFM
aggressive: AG
alpha-1-acid glycoprotein 1: ORM1
alpha-1-antitrypsin: SERPINA1
area under curve: AUC
attractin: ATRN
carboxypeptidase E: CPE
CD63 antigen: CD63
CD97 antigen: CD97
clusterin: CLU
data independent acquisition: DIA
desmocollin-2: DSC2
digital rectal examination: DRE
Food and Drug Administration: FDA
kallikrein-11: KLK11
leucine-rich alpha-2-glycoprotein: LRG1
mass spectrometry: MS
mix-mode anion exchange: MAX
neuroplastin: NPTN
non-aggressive: NAG
non-secretory ribonuclease: RNASE2
pancreatic secretory granule membrane major glycoprotein GP2: GP2
Progranulin: GRN
prostaglandin-H2 D-isomerase: PTGDS
prostate cancer antigen-3: PCA3
prostate cancer: PCa
prostate-specific antigen: PSA
prostatic acid phosphatase: ACPP
protein-lysine 6-oxidase: LOX
receptor-type tyrosine-protein phosphatase N2: PTPRN2
transmembrane serine protease 2: TMPRSS2
uromodulin: UMOD
